# Slow progressors to type 1 diabetes lose islet autoantibodies over time, have few islet antigen-specific CD8^+^ T cells and exhibit a distinct CD95^hi^ B cell phenotype

**DOI:** 10.1007/s00125-020-05114-7

**Published:** 2020-03-10

**Authors:** Stephanie J. Hanna, Wendy E. Powell, Anna E. Long, Emma J. S. Robinson, Joanne Davies, Clare Megson, Alexandra Howell, Taz J. Jones, Kristin Ladell, David A. Price, Colin M. Dayan, Alistair J. K. Williams, Kathleen M. Gillespie, F. Susan Wong

**Affiliations:** 1 Division of Infection and Immunity, Cardiff University School of Medicine, Heath Park, Cardiff, CF14 4XN UK; 2 Diabetes and Metabolism, Bristol Medical School, University of Bristol, Bristol, UK

**Keywords:** Autoantibodies, B cells, CD95, DcR3, ELISpot, Peptide-HLA class I tetramers, Proinsulin, Slow progressors to type 1 diabetes, T cells

## Abstract

**Aims/hypothesis:**

The aim of this study was to characterise islet autoantibody profiles and immune cell phenotypes in slow progressors to type 1 diabetes.

**Methods:**

Immunological variables were compared across peripheral blood samples obtained from slow progressors to type 1 diabetes, individuals with newly diagnosed or long-standing type 1 diabetes, and healthy individuals. Polychromatic flow cytometry was used to characterise the phenotypic attributes of B and T cells. Islet autoantigen-specific B cells were quantified using an enzyme-linked immunospot (ELISpot) assay and islet autoantigen-specific CD8^+^ T cells were quantified using peptide–HLA class I tetramers. Radioimmunoassays were used to detect islet autoantibodies. Sera were assayed for various chemokines, cytokines and soluble receptors via ELISAs.

**Results:**

Islet autoantibodies were lost over time in slow progressors. Various B cell subsets expressed higher levels of CD95 in slow progressors, especially after polyclonal stimulation, compared with the corresponding B cell subsets in healthy donors (*p* < 0.05). The phenotypic characteristics of CD4^+^ and CD8^+^ T cells were similar in slow progressors and healthy donors. Lower frequencies of CD4^+^ T cells with a central memory phenotype (CD27^int^, CD127^+^, CD95^int^) were observed in slow progressors compared with healthy donors (mean percentage of total CD4^+^ T cells was 3.00% in slow progressors vs 4.67% in healthy donors, *p* < 0.05). Autoreactive B cell responses to proinsulin were detected at higher frequencies in slow progressors compared with healthy donors (median no. of spots was 0 in healthy donors vs 24.34 in slow progressors, *p* < 0.05) in an ELISpot assay. Islet autoantigen-specific CD8^+^ T cell responses were largely absent in slow progressors and healthy donors. Serum levels of DcR3, the decoy receptor for CD95L, were elevated in slow progressors compared with healthy donors (median was 1087 pg/ml in slow progressors vs 651 pg/ml in healthy donors, *p* = 0.06).

**Conclusions/interpretation:**

In this study, we found that slow progression to type 1 diabetes was associated with a loss of islet autoantibodies and a distinct B cell phenotype, consistent with enhanced apoptotic regulation of peripheral autoreactivity via CD95. These phenotypic changes warrant further studies in larger cohorts to determine their functional implications.

**Electronic supplementary material:**

The online version of this article (10.1007/s00125-020-05114-7) contains peer-reviewed but unedited supplementary material, which is available to authorised users.

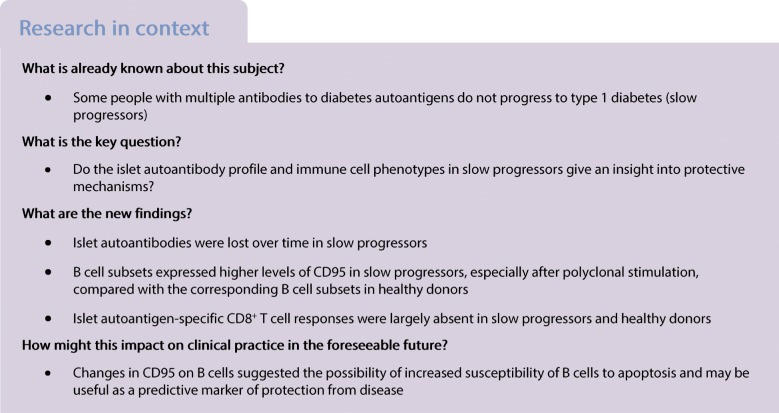



## Introduction

The presence of autoantibodies specific for two or more islet antigens is a reliable predictor of progression to type 1 diabetes. Longitudinal studies of at-risk individuals have shown, however, that up to 30% of people positive for multiple autoantibodies do not progress to type 1 diabetes within 10 years [1]. Such individuals were recruited to the Slow or Non-progressive Autoimmunity to the Islets of Langerhans (SNAIL) study from five other cohort studies, including the Bart’s Oxford Family Study (BOX) [2]. All four major autoantibodies, specific for GAD (GADA), islet antigen-2 (IA-2A), insulin (IAA) and zinc transporter 8 (ZnT8A), were found in these slow progressors to type 1 diabetes (hereafter referred to as ‘slow progressors’). The frequency of HLA class II risk alleles in this cohort was similar to that observed in children diagnosed with type 1 diabetes over the age of 10 years. Other factors may therefore be responsible for non-progression, and the identification of protective mechanisms could inform future preventive therapies.

People with type 1 diabetes display altered frequencies and function of a number of lymphocyte subsets, as well as different expression levels of surface markers; frequency and suppressive ability of Tregs are reduced in type 1 diabetes [3]. Expression of C-X-C motif chemokine receptor 3 (CXCR3) is reduced on CD3^+^ T cells in individuals with long-standing type 1 diabetes [4–6]. Although beta cell-autoreactive CD8^+^ T cells are found in healthy volunteers, these CD8^+^ T cells from people with type 1 diabetes have a more differentiated phenotype that includes T_SCM_ [7]. Reported perturbations among B cell subsets in type 1 diabetes include increased frequencies of transitional (CD27^−^IgD^+^IgM^−^) B cells [8], increased frequencies of marginal zone (CD19^+^CD21^+^CD23^−^) B cells and decreased frequencies of follicular (CD19^+^CD21^−^CD23^+^) and regulatory (CD1d^+^CD5^+^CD19^+^) B cells [9]. Furthermore, there are decreased frequencies of other putative regulatory (CD24^++^CD38^++^) B cells [10] and decreased frequencies of CD40^+^IL10^+^ B cells [11]. In addition, high-affinity insulin-binding B cells are lost from the peripheral blood of people with newly diagnosed type 1 diabetes, along with other anergic B cells, and these return in people with long-standing type 1 diabetes [12]. Some individuals with type 1 diabetes also exhibit reduced frequencies of circulating B cells that express CD95 (FasR) and transmembrane activator and CAML receptor (TACI) [13]. We have recently found that B cells from people with long-standing diabetes display reduced expression of CXCR3, CD95, B220 and CD24 [6].

The SNAIL study participants may also have biomarkers determining which autoantibody-positive individuals are protected from development of diabetes. In this study, we characterised the immunological profiles of slow progressors recruited to the BOX study (*n* = 10), people with newly diagnosed or long-standing type 1 diabetes (*n* = 18 in each group), and healthy donors (*n* = 23). In addition, for the HLA class I-A2^+^ individuals, we also assessed the ability of the CD8^+^ T cells to recognise autoantigenic peptides, presented by HLA-A2–peptide tetramers (*n* = 5 for healthy donors, 7 for slow progressors, 9 for newly diagnosed diabetes and 10 for long-standing diabetes). Finally, we analysed serum cytokine and chemokine expression, comparing slow progressors with healthy donors, and people with newly diagnosed and long-standing type 1 diabetes.

## Methods

### Participants

The BOX study is a longitudinal study examining risk factors for type 1 diabetes in parents or siblings of probands diagnosed under the age of 21 [14]. Between 1985 and 2012, islet autoantibodies were tested in at least one sample from 5881 relatives who were free from diabetes at the time of testing. Slow progressors were defined as people, initially seropositive for at least two islet-specific autoantibodies, who remained free from diabetes for at least 10 years.

Of 36 slow progressors identified in the BOX study, confirmed as multiple islet autoantibody positive using current harmonised assays [2], 19 were not included in the current analysis: 11 were excluded because they developed diabetes more than 10 years after the initial serum sample (and no follow-up serum sample was available after the 10 year point before they had developed diabetes); seven were lost to follow-up; and one had chronic lymphocytic leukaemia.

**Fig. 1:**
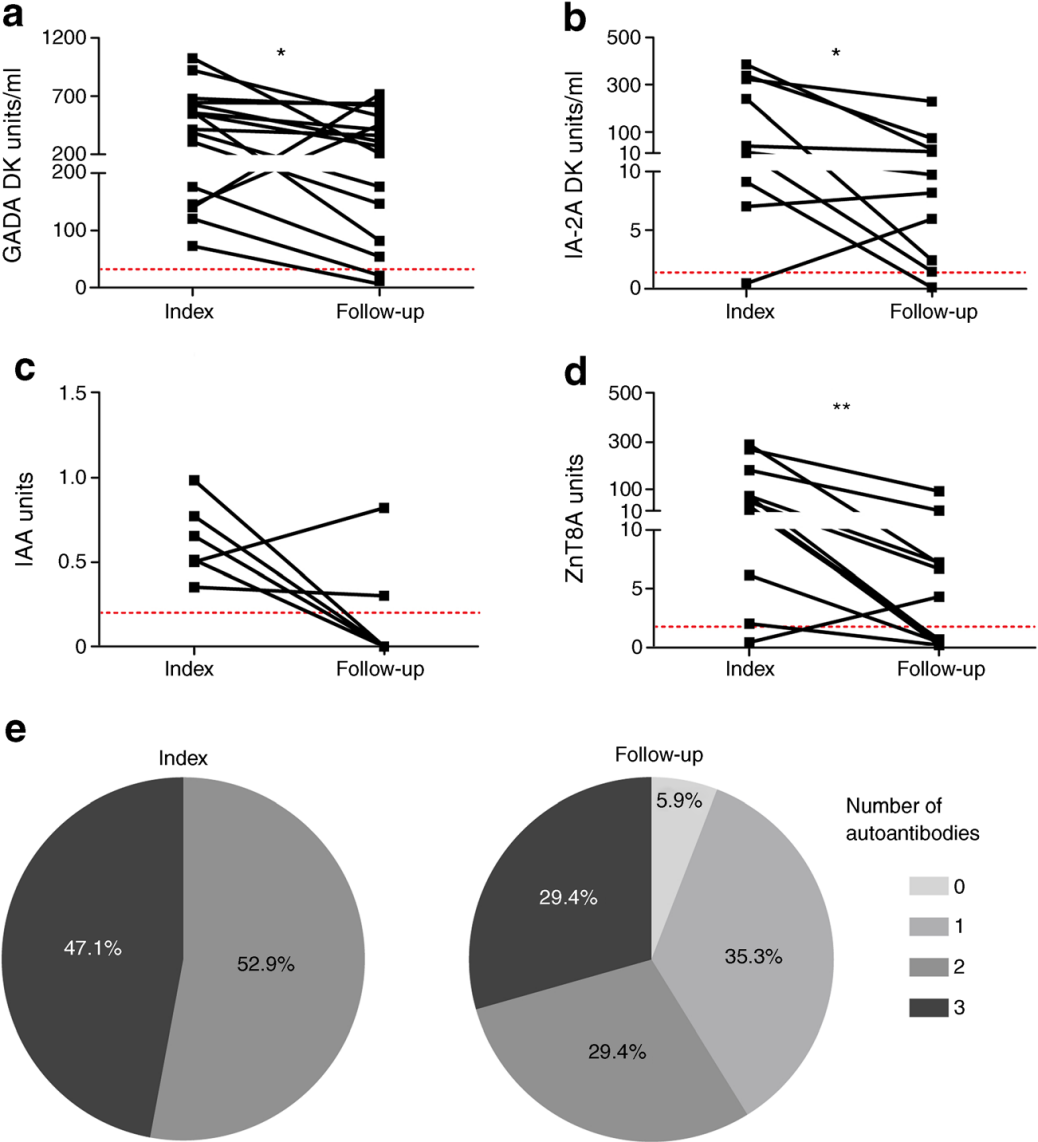
Loss of autoantibodies in some slow progressors over time. Islet autoantibody titres were measured in follow-up samples obtained from slow progressors (*n* = 17) at least 10 years after initial testing (index) as part of the BOX study. Follow-up samples were acquired at the time of sampling for immune cell analysis (*n* = 10), before/at diagnosis (*n* = 3), or as recently as possible (*n* = 4). (**a**–**d**) Only titres for individuals positive for a specific autoantibody are shown: (**a**) GADA (*n* = 16); (**b**) IA-2A (*n* = 10); (**c**) IAA (*n* = 6); (**d**) ZnT8A (*n* = 12). Dotted lines indicate the threshold for seropositive islet autoantibody titres: GADA ≥33 DK units/ml; IA-2A ≥1.4 DK units/ml; IAA ≥0.2 units; ZnT8A ≥1.8 units. GADA and IA-2A titres are expressed in DK units/ml derived from a standard curve developed for the NIDDK-sponsored Islet Autoantibody Harmonization Program. IAA and ZnT8A titres are expressed in units derived from in-house standard curves, measured in duplicate in 5 μl and 2 μl of serum respectively. *p < 0.05 and **p < 0.01 for follow-up vs index (determined using the Wilcoxon matched pairs test). (**e**) Proportion of individuals with 0, 1, 2 or 3 autoantibodies (GADA, IA-2A, IAA and/or ZnT8A); *p* = 0.007 determined using Fisher’s exact test comparing groups of ≥2 and <2 autoantibodies for index vs follow-up samples

For this islet-specific autoantibody analysis, we examined 17 slow progressors with a first multiple islet autoantibody sample available and a follow-up sample taken more than 10 years later (Fig. 1). Subsequently, a further three slow progressors developed diabetes, one was lost to follow-up and three declined further sampling. The remaining ten slow progressors provided large-volume blood samples for the immunology studies described here. The consort diagram is illustrated in electronic supplementary material (ESM) Fig. 1.

We recruited sex-matched healthy donors of similar age, seronegative for islet-specific autoantibodies, with no family history of type 1 diabetes or other autoimmune conditions.

Adults with newly diagnosed or long-standing type 1 diabetes were also recruited, to provide context for the similarities or differences observed between healthy donors and slow progressors. Type 1 diabetes was diagnosed according ADA criteria [15] and insulin treatment was commenced within 1 month of diagnosis. Time from diagnosis was categorised as <1 year for newly diagnosed individuals and >3 years for individuals with long-standing diabetes. Cohort details are summarised in ESM Table 1.

### Ethics

The BOX study was approved by the South Central–Oxford C National Research Ethics Committee. Study of progressors and control individuals was approved by the South East Wales Research Ethics Committee and conducted in accordance with the principles of Good Clinical Practice established by the International Council for Harmonization/WHO. All participants provided written informed consent prior to enrolment, as mandated by the Declaration of Helsinki.

### Islet-specific autoantibodies

GADA, IA-2A, IAA and ZnT8A were measured using fully validated radioimmunoassays [16]. The higher value from two distinct assays, one for the R325 variant and one for the W325 variant, was reported for ZnT8A. Titres of IAA and ZnT8A, measured in duplicate in 5 μl and 2 μl of serum respectively, were expressed in units derived from in-house standard curves. Titres of GADA and IA-2A were expressed in digestive and kidney (DK) units/ml derived from a standard curve developed for the NIDDK-sponsored Islet Autoantibody Harmonization Program [17].

### HLA class II genotyping

Genomic DNA was extracted from whole-blood samples collected in EDTA. HLA class II *DRB1* alleles were resolved using polymerase chain reactions with sequence-specific primers [18].

### Blood samples

Peripheral blood mononuclear cells (PBMCs) were isolated from heparinised samples of whole blood via density gradient centrifugation over Lymphoprep (Stem Cell Technologies, Cambridge, UK). Aliquots of 5 × 10^6^ to 20 × 10^6^ PBMCs/ml per vial were stored in liquid nitrogen after cooling overnight to −80°C at a controlled rate of −1°C/min in a Cryostor CS10 (Sigma-Aldrich, St Louis, MO, USA).

### Tetramers

Fluorochrome-labelled peptide-HLA-A2 tetramers were assembled from monomers from NIH Tetramer Core Facility (Atlanta, GA, USA) and streptavidin Qdots (Thermo Fisher Scientific, Waltham, MA, USA). Tetramer staining was performed as described previously [19]. Briefly, thawed PBMCs were treated with 50 nmol/l dasatinib for 15 min at 37°C, then pelleted and resuspended in tetramer Qdot master mix for 15 min at 37°C (details in ESM Table 2). Cells were incubated with LIVE/DEAD Fixable Aqua (Thermo Fisher Scientific) for 10 min at room temperature and stained for 30 min at 4°C with titrated concentrations of the following antibodies: anti-CD8–AF700 (clone RPA-T8) (BD Biosciences, San Jose, CA, USA); anti-CD4–FITC (clone OKT4); anti-CD14–FITC (clone 61D3); anti-CD16–FITC (clone eBioCB16); anti-CD20–FITC (clone 2H7); and anti-CD40–FITC (clone 5C3) (dump channel; eBioscience, San Diego, CA, USA). Data were acquired using a modified FACSAria II flow cytometer (BD Biosciences). All flow cytometry data were analysed with FlowJo software version 10 (Tree Star, Ashland, OR, USA).

### T cell panel

Thawed PBMCs were blocked with TruStain (BioLegend, San Diego, CA, USA) for 5 min at room temperature, incubated with LIVE/DEAD Fixable Aqua (Thermo Fisher Scientific) for 10 min at room temperature and stained for 30 min at 4°C with titrated concentrations of the following antibodies: (1) anti-CD3–APC/Fire750 (clone SK7), anti-CD8a–BV711 (clone RPA-T8), anti-CD57–PE-Cy7 (clone HNK-1), anti-CD95–PE-Cy5 (clone DX2) and anti-PD-1–BV421 (clone EH12.2H7) (BioLegend); (2) anti-CD45RA–ECD (clone 2H4LDH11LDB9) and anti-CD127–PE (clone R34.34) (Beckman Coulter, Brea, CA, USA); (3) anti-CD14–V500 (clone MSE2) and anti-CD19–V500 (clone HIB19) (BD Biosciences); (4) anti-CD4–PE-Cy5.5 (clone S3.5) and anti-CD27–Qdot 605 (clone CLB-27/1) (Thermo Fisher Scientific); and (5) anti-CXCR3–FITC (clone 49801) (R&D Systems, Minneapolis, MN, USA). Cells were then washed in PBS containing 0.5% wt/vol. BSA and 2 mmol/l EDTA, fixed with 1% wt/vol. paraformaldehyde and acquired using a modified FACSAria II flow cytometer (BD Biosciences).

### B cell panel

Thawed PBMCs were blocked with TruStain (BioLegend) for 5 min at room temperature, incubated with LIVE/DEAD Fixable Aqua (Thermo Fisher Scientific) for 10 min at room temperature and stained for 30 min at 4°C with titrated concentrations of the following antibodies: (1) anti-CD19–PE-Cy7 (clone SJ25C1) and anti-CD24–APC-eFluor780 (clone SN3) (eBioscience); (2) anti-CD3–BV711 (clone OKT3), anti-CD45R/B220–BV421 (clone RA3-6B2) and anti-IgD–AF488 (clone IA6-2) (BioLegend); (3) anti-CD27–Qdot 605 (clone CLB-27/1) (Thermo Fisher Scientific); (4) anti-CD21–PE-Cy5 (clone B-ly4), anti-CD38–PE-CF594 (clone HIT2) and anti-CXCR3–PE (clone IC6/CXCR3) (BD Biosciences); and (5) anti-CD95–APC (clone DX2) (Miltenyi Biotec, Bergisch Gladbach, Germany). Cells were then washed in PBS containing 0.5% wt/vol. BSA and 2 mmol/l EDTA, fixed with 1% wt/vol. paraformaldehyde, and acquired using a modified FACSAria II flow cytometer (BD Biosciences) or an LSR Fortessa (for stimulated B cells).

### B cell stimulation

Freshly isolated PBMCs were cultured with 0.5 μmol/l CpG oligodeoxynucleotide 2006 (Eurofin Genomics, Ebersberg, Germany), 0.5 μg/ml protein-A soluble from *Staphylococcus aureus* Cowan strain (Sigma-Aldrich) and 1 μg/ml pokeweed mitogen (Sigma-Aldrich) in 10% heat-inactivated AB serum/RPMI (stimulated) or with 10% heat-inactivated AB serum/RPMI alone (unstimulated) for 5 days at 37°C.

### B cell enzyme-linked immunospot assay

For the enzyme-linked immunospot (ELISpot) assay, multiScreen-IP filter plates (Merck Millipore, Burlington, MA, USA) were pre-wetted with 70% molecular grade ethanol (Sigma-Aldrich), washed and coated with PBS, 0.5 μg/ml goat anti-human IgG (Newmarket Scientific, Newmarket, UK), 0.8 or 1.6 μg/ml GAD (RSR, Dallas, TX, USA), 0.8 or 1.6 μg/ml islet antigen-2 (IA-2) (RSR), or 10 or 50 μg/ml proinsulin (Biomm, Nova Lima, Brazil) overnight at 4°C. Plates were washed and blocked with 10% heat-inactivated FBS/RPMI overnight at 4°C. Stimulated cells were washed in 10% heat-inactivated FBS/RPMI, resuspended at 5 × 10^5^ cells/ml (for the IgG positive control) or 4 × 10^6^ cells/ml (for all other conditions), and plated at 100 μl/well (*n* ≥ 6 wells per condition). Plates were incubated for 5 h at 37°C, washed in 0.05% Tween 20/PBS and coated with 0.5 μg/ml goat anti-human IgG Fc biotin in 5% heat-inactivated FBS/PBS overnight at 4°C. Antibody secretion was revealed using ExtrAvidin (Sigma-Aldrich) followed by BCIP/NBT substrate (Sigma-Aldrich). Plates were washed and dried overnight and spots were counted using a BIO-SYS Bioreader 4000 (BioSys, Miami, FL, USA). The PBS background was subtracted from all counts (mean±SD background 142 ± 75, range 30.5–245, not significantly different between healthy donors and slow progressors).

### Spanning-tree progression analysis of density-normalised events

Live T cells were identified in serial gates as singlets, lymphocytes, LIVE/DEAD Fixable Aqua^−^, CD19^−^, CD3^+^ events and gated as CD4^+^, CD8^+^, CD4/CD8 double-positive, or CD4/CD8 double-negative cells using FlowJo. Compensated flow cytometry files were exported for Spanning-tree Progression Analysis of Density-normalized Events (SPADE) using fixed settings (K-means algorithm, 100 clusters, Arcsinh transformation with cofactor 150) in SPADE software version 3.0 [6, 20, 21]. Expression of cell surface markers (CD3, CD4, CD8, CD27, CD57, CD95, CD45RA, CD127, CXCR3 and programmed cell-death protein 1 [PD-1]) was assessed. Live B cells were identified in serial gates as singlets, lymphocytes, LIVE/DEAD Fixable Aqua^−^, CD3^−^, CD19^+^ events using FlowJo. Data were processed as above with the same settings in SPADE. Expression of cell surface markers (B220, CD19, CD21, CD24, CD27, CD38, CD95, CXCR3 and IgD) was assessed. Auto-partitioning was used to divide the resulting trees into eight areas. Median expression of each marker was calculated for each donor in each node.

### Serum chemokines, cytokines and soluble receptors

Serum samples were analysed using the Meso Scale Discovery U-PLEX platform to quantify CXCL10, CXCL11, IL-4, IL-6, IL-10 and IFN-γ (MSD, Rockville, MD, USA) or DuoSet ELISA Kits to quantify B cell activating factor (BAFF), CD95L (FasL), CXCL9, Decoy receptor 3 (DcR3) and TGF-β (R&D Systems).

### Statistics

Differences in mean values between two groups were compared using an unpaired Student’s *t* test (if the data were normally distributed) or the Mann–Whitney *U* test (if the data were not normally distributed). Immune cell variables were compared primarily between slow progressors and healthy donors. Newly diagnosed and long-standing type 1 diabetes samples were included to add context but were not included in statistical analysis due to small sample sizes and lack of age matching between newly diagnosed individuals and slow progressors. Therefore, no statistical comparisons were made between people with type 1 diabetes and either healthy donors or slow progressors. The exception to this was the peptide-HLA-A2 tetramer study, where people with type 1 diabetes were used as a positive control to validate the assay and results were compared between people with type 1 diabetes and healthy donors using a Mann–Whitney *U* test. Individual autoantibody titres at different time-points were compared using the Wilcoxon matched pairs test. The number of autoantibodies with elevated titres was compared across time-points using Fisher’s exact test. All statistical tests were performed using Prism software version 6 (GraphPad, San Diego, CA, USA).

## Results

### Islet autoantibody profiles show that some slow progressors lose autoantibodies over time

Slow progressors were initially seropositive for at least two islet autoantibodies, the titres of which decreased over time in most people (GADA *n* = 16, *p* = 0.029; IA-2A *n* = 10, *p* = 0.014; IAA *n* = 6, *p* = 0.094; ZnT8A *n* = 12, *p* = 0.002) (Fig. 1a–d). Of the 17 slow progressors included in longitudinal islet autoantibody analysis in this study, four (24%) did not lose positivity for any islet autoantibody although in general the titre decreased over time. Of the remainder, we observed and confirmed loss of IAA four times, ZnT8A twice, IA-2A once and GADA once. However, antibody loss was not confirmed in four individuals with ZnT8A and one individual with GADA because these occurred in the most recent sample. Overall, multiple antibody positivity decreased (*p* = 0.007) (Fig. 1e). In the cohort of slow progressors analysed for immune cell variables (*n* = 10), the number of autoantibodies with titres considered seropositive was either reduced from three to two (*n* = 1) or from two to one (*n* = 3) in the test samples (the latter indicated by red squares in subsequent figures), or remained unchanged relative to the index sample (*n* = 6) (ESM Table 1).

### Slow progressors do not show islet autoantigen-specific CD8^+^ T cell responses

**Fig. 2:**
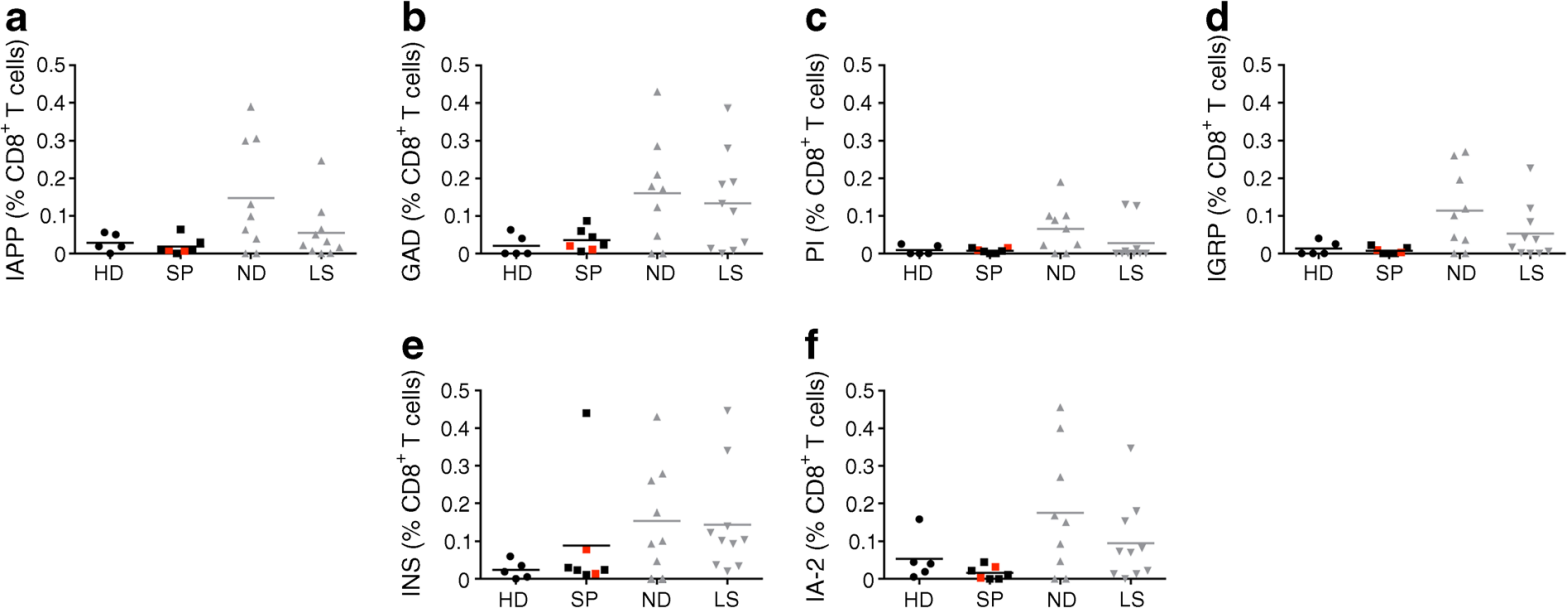
Islet autoantigen-specific CD8^+^ T cell responses are not found in slow progressors. HLA-A2^+^ participants were tested for CD8^+^ T cell responses to known islet autoantigens using peptide–HLA-A2 tetramers (ESM Table 2). Background staining was determined using a non-interfacing peptide–HLA-A2 tetramer. Results are shown as background-subtracted frequencies among CD8^+^ T cells. Tetramer responses are shown to: (**a**) islet amyloid polypeptide (KLQVFLIVL); (**b**) GAD (VMNILLQYVV); (**c**) proinsulin (ALWGPDPAAA); (**d**) islet-specific glucose-6-phosphatase catalytic subunit-related protein (VLFGLGFAI); (**e**) insulin (HLVEALYLV); (**f**) IA-2 (MVWESGCTV). Red squares denote slow progressors who tested seropositive for a single islet autoantibody at the time of immune cell analysis. Horizontal lines indicate median values. Results for people with newly diagnosed and long-standing type 1 diabetes are shown in grey to add context and to represent a positive control in the assay but were not included in the statistical analysis shown on the graph due to small sample sizes and lack of age matching between newly diagnosed and slow-progressing donors. HD, healthy donors; IA-2, insulin antigen-2; IAPP, islet amyloid polypeptide; IGRP, islet-specific glucose-6-phosphatase catalytic subunit-related protein; INS, insulin; LS, long-standing type 1 diabetes; ND, newly diagnosed type 1 diabetes; PI, proinsulin; SP, slow progressors

HLA-A2^+^ participants were tested for CD8^+^ T cell responses to known islet autoantigens using peptide–HLA-A2 tetramers (ESM Table 2). Minimal islet autoantigen-specific CD8^+^ T cell responses were observed in slow progressors and healthy donors (Fig. 2a–f and ESM Fig. 2). In contrast, individuals with newly diagnosed type 1 diabetes harboured significantly higher frequencies of CD8^+^ T cells specific for epitopes derived from GAD (*p* < 0.05), proinsulin (*p* < 0.05), islet-specific glucose-6-phosphatase catalytic subunit-related protein (*p* < 0.05) and insulin (*p* < 0.05) compared with healthy donors (Fig. 2b–e), and individuals with long-standing type 1 diabetes harboured significantly higher frequencies of CD8^+^ T cells specific for the epitopes derived from GAD (*p* < 0.05) and insulin (*p* < 0.05) compared with healthy donors (Fig. 2b,e).

### T cells from slow progressors show decreased CD95 expression and a decreased percentage of central memory T cells

**Fig. 3:**
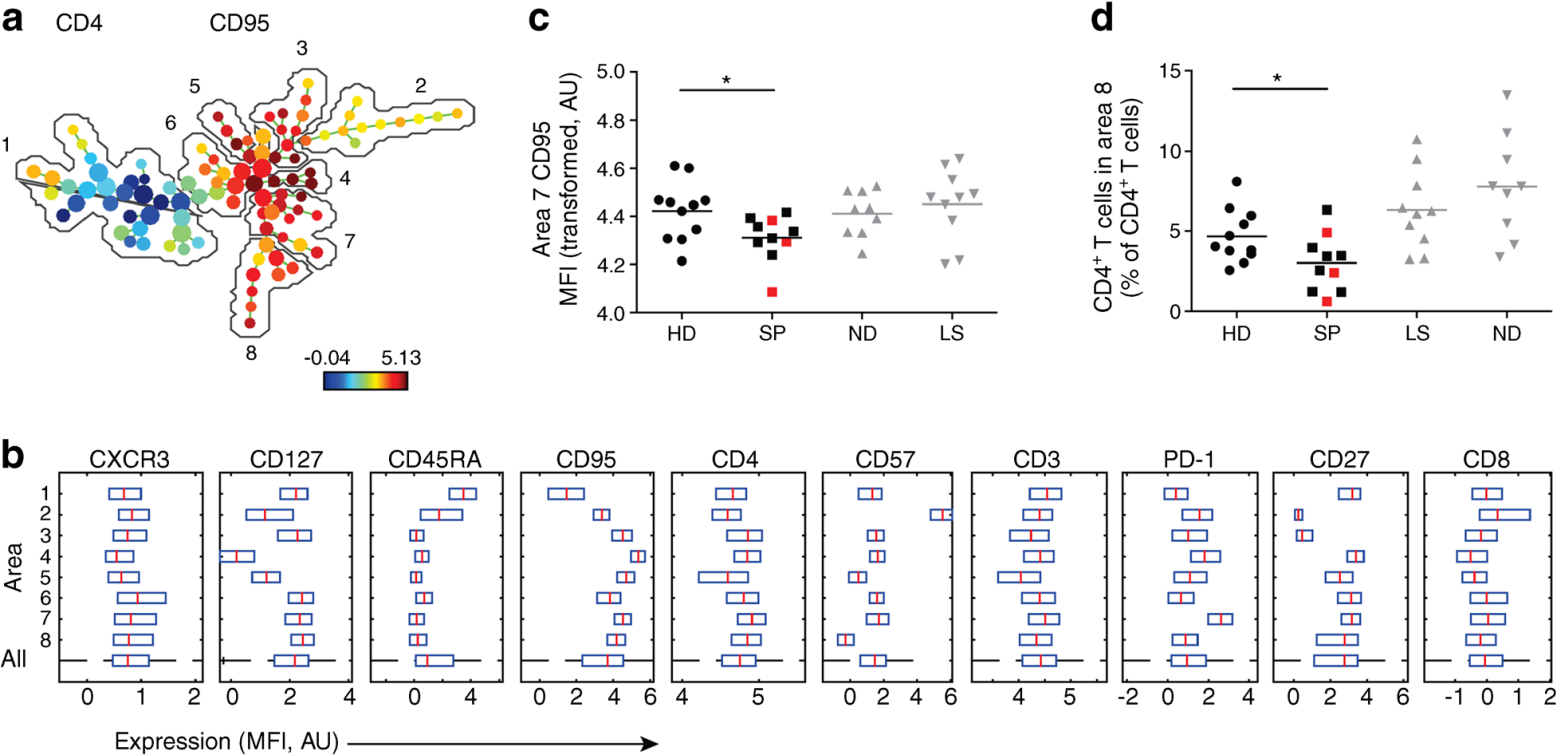
Phenotypic characteristics of T cells in slow progressors indicate decreased CD95 expression and a decreased percentage of central memory T cells. Expression of the indicated markers was assessed using flow cytometry. (**a**) SPADE image of pooled CD4^+^ T cells from all participants, auto-partitioned into eight annotated areas with node size scaled to the log number of cells in each node, showing median CD95 expression as a heatmap. Based on the expression of CD27 and CD45RA, the cells in the different areas were designated as follows: area 1, naive (CD27^+^CD45RA^+^); area 2, effector (CD27^−^CD45RA^int^); area 3, effector memory (CD27^−^CD45RA^−^); areas 4–8, memory (CD27^int/+^CD45RA^−^). (**b**) SPADE boxplots showing marker distribution in each area or in all areas for the pooled CD4^+^ T cell samples depicted in (**a**). Central red lines indicate median values and the ends of blue boxes indicate interquartile ranges. The dashed horizontal line at the bottom indicates the ‘All’ category. (**c**) CD95 expression (transformed values) in CD4^+^ T cell area 7 for each participant. (**d**) Percentage of CD4^+^ T cells in CD4^+^ T cell area 8 for each participant. In (**c**) and (**d**), horizontal lines indicate mean values and red squares denote slow progressors who tested seropositive for a single islet autoantibody at the time of immune cell analysis. *p < 0.05 for slow progressors vs healthy donors (determined using an unpaired Student’s *t* test). Results for people with newly diagnosed and long-standing type 1 diabetes are shown in grey to add context but were not included in statistical analysis due to small sample sizes and lack of age matching between newly diagnosed and slow-progressing individuals. AU, arbitrary unit; HD, healthy donors; LS, long-standing type 1 diabetes; MFI, median fluorescence intensity; ND, newly diagnosed type 1 diabetes; SP, slow progressors

Expression of the following cell surface markers was assessed using flow cytometry: CD3, CD4, CD8, CD27, CD57, CD95, CD45RA, CD127, CXCR3 and PD-1. Viable CD3^+^ cells were gated as CD4^+^, CD8^+^, double-positive or double-negative events and exported for multivariate analysis in SPADE (Fig. 3a–d). The resulting SPADE tree was auto-partitioned into eight annotated areas and the cells that were in each area were identified based on the expression of CD27, IgD and additional markers. Slow progressors exhibited decreased expression of CD95 among CD4^+^ T cells with a transitional memory phenotype (area 7; CD27^+^ CD45RA^−^CD127^+^PD-1^++^) compared with healthy donors (*p* < 0.05) (Fig. 3c). Lower frequencies of CD4^+^ T cells with a central memory phenotype (area 8; CD27^int^CD45RA^−^CD127^+^CD95^int^) were also present in slow progressors compared with healthy donors (mean percentage of total CD4^+^ T cells was 4.67% in healthy donors vs 3.00% in slow progressors, *p* < 0.05) (Fig. 3d). These differences were not observed in individuals with newly diagnosed or long-standing type 1 diabetes. Moreover, higher frequencies of CD4^+^ T cells with a central memory phenotype (CD27^int^CD127^+^CD95^int^) were detected in individuals with newly diagnosed type 1 diabetes (mean percentage of total CD4^+^ T cells was 7.78%, *p* < 0.05) compared with healthy donors (Fig. 3d). Representative flow cytometric plots of analogous populations of cells, manually gated in FlowJo are shown in ESM Fig. 3.

### B cells from slow progressors show increased expression of CD95

**Fig. 4:**
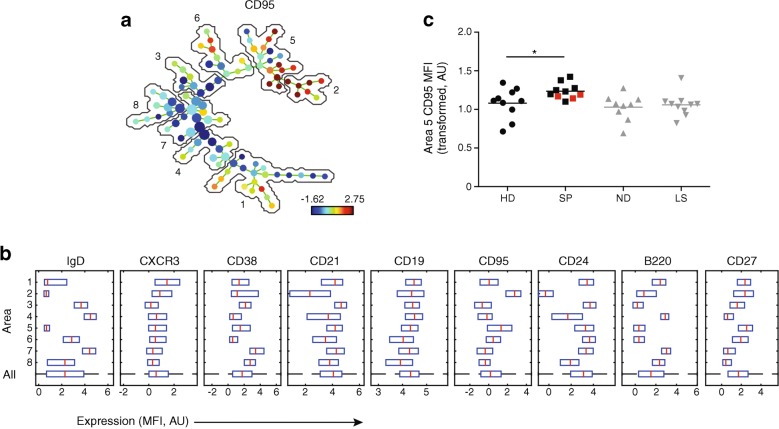
Phenotypic characterisation of unstimulated B cells in slow progressors demonstrate increased expression of CD95 among B cells with a switched memory phenotype. Expression of the indicated markers in unstimulated samples was assessed using flow cytometry. (**a**) SPADE image of pooled B cells from all participants, auto-partitioned into eight annotated areas with node size scaled to the log number of cells in each node, showing median CD95 expression as a heatmap. Based on the expression of CD27 and IgD, the cells in the different areas were designated as follows: areas 1, 2 and 5, switched memory (CD27^+^IgD^−^); area 3, unswitched (CD27^int^IgD^+^); areas 4 and 7, naive (CD27^−^IgD^+^); area 6, transitional (CD27^int^IgD^int^); area 8, naive/switched (CD27^−^IgD^int^). (**b**) SPADE boxplots showing marker distribution in each area or in all areas for the pooled samples depicted in (**a**). Central red lines indicate median values and the ends of blue boxes indicate interquartile ranges. The dashed horizontal line at the bottom indicates the ‘All’ category. (**c**) CD95 expression (transformed values) in area 5 for each participant. Horizontal lines indicate mean values and red squares denote slow progressors who tested seropositive for a single islet autoantibody at the time of immune cell analysis. *p < 0.05 for slow progressors vs healthy donors (determined using an unpaired Student’s *t* test). Results for people with newly diagnosed and long-standing type 1 diabetes are shown in grey to add context but were not included in statistical analysis due to small sample sizes and lack of age matching between newly diagnosed and slow-progressing individuals. AU, arbitrary unit; HD, healthy donors; LS, long-standing type 1 diabetes; MFI, median fluorescence intensity; ND, newly diagnosed type 1 diabetes; SP, slow progressors

Expression of the following cell surface markers was assessed using flow cytometry: B220, CD19, CD21, CD24, CD27, CD38, CD95, CXCR3 and IgD. Viable CD3^−^CD19^+^ cells were gated and exported for multivariate analysis in SPADE (Fig. 4a–c). First, we analysed the phenotype of the B cells from uncultured PBMCs. All samples were thawed and analysed on the same day using a panel of B cell phenotyping antibodies and SPADE. When compared with healthy donors, the long-standing and newly diagnosed diabetes cohorts had a number of phenotypic differences in the B cell compartment. These included a decrease in CXCR3 expression, as we previously reported [6]. No such changes were observed in the corresponding B cell subsets in slow progressors. However, slow progressors exhibited increased expression of CD95 among B cells with a switched memory phenotype (CD27^+^, IgD^−^) compared with healthy donors (*p* < 0.05), in contrast to individuals with newly diagnosed or long-standing type 1 diabetes (Fig. 4c). Representative flow cytometric plots of analogous populations of cells manually gated in FlowJo are shown in ESM Fig. 4.

**Fig. 5:**
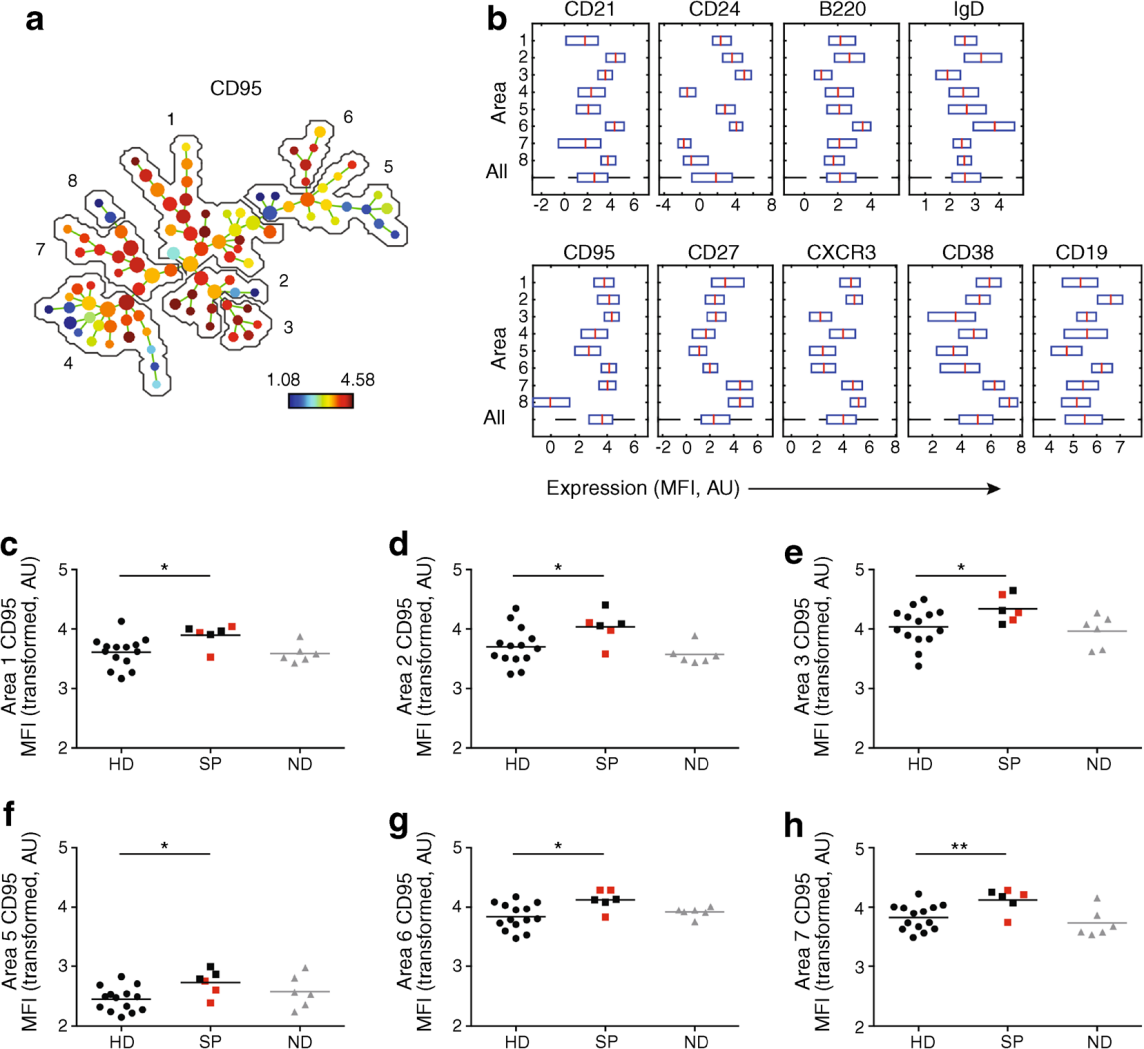
Phenotypic characteristics of stimulated B cells in slow progressors show increased CD95 expression compared with cells from healthy donors. Expression of the indicated markers in stimulated samples was assessed using flow cytometry. (**a**) SPADE image of pooled B cells from all participants, auto-partitioned into eight annotated areas with node size scaled to the log number of cells in each node, showing median CD95 expression as a heatmap. Based on the expression of CD27 and IgD, the cells in the different areas were designated as follows: area 1, switched memory (CD27^+^IgD^−^); area 2, memory (CD27^int^IgD^int^); area 3, switched memory (CD27^int^IgD^−^); areas 4 and 5, switched (CD27^−^IgD^−^); area 6, naive (CD27^−^IgD^+^); areas 7 and 8, switched memory (CD27^++^IgD^−^). (**b**) SPADE boxplots of marker distribution in each area or in all areas for the pooled samples depicted in (**a**). Central red lines indicate median values and the ends of blue boxes indicate interquartile ranges. The dashed horizontal line at the bottom indicates the ‘All’ category. (**c**–**h**) CD95 expression (transformed values) in areas 1 (**c**), 2 (**d**), 3 (**e**), 5 (**f**), 6 (**g**) and 7 (**h**) for each participant. In (**c**–**h**), horizontal lines indicate mean values and red squares denote slow progressors who tested seropositive for a single islet autoantibody at the time of immune cell analysis. *p < 0.05 and **p < 0.01 for slow progressors vs healthy donors (determined using an unpaired Student’s *t* test). Results for people with newly diagnosed type 1 diabetes are shown in grey to add context but were not included in statistical analysis due to small sample sizes and lack of age matching between newly diagnosed and slow-progressing individuals. AU, arbitrary unit; HD, healthy donors; MFI, median fluorescence intensity; ND, newly diagnosed type 1 diabetes; SP, slow progressors

To extend these findings, we conducted parallel experiments with stimulated B cells from slow progressors (*n* = 6), individuals with newly diagnosed type 1 diabetes (*n* = 6) and healthy donors (*n* = 14) (Fig. 5a–h), prepared from freshly isolated PBMCs. Slow progressors exhibited increased expression of CD95 among various B cell subsets when compared with healthy donors: area 1, switched memory (CD27^+^IgD^−^); area 2, memory (CD27^int^IgD^int^); area 3, switched memory (CD27^int^IgD^−^); area 5, switched (CD27^−^IgD^−^); area 6, naive (CD27^−^IgD^+^) and area 7, switched memory (CD27^++^IgD^−^); *p* < 0.05). Cells from individuals with newly diagnosed type 1 diabetes showed no such changes (Fig. 5c–h and ESM Fig. 5).

### Slow progressors show increased B cell responses to proinsulin but not to GAD or insulin antigen-2

**Fig. 6:**
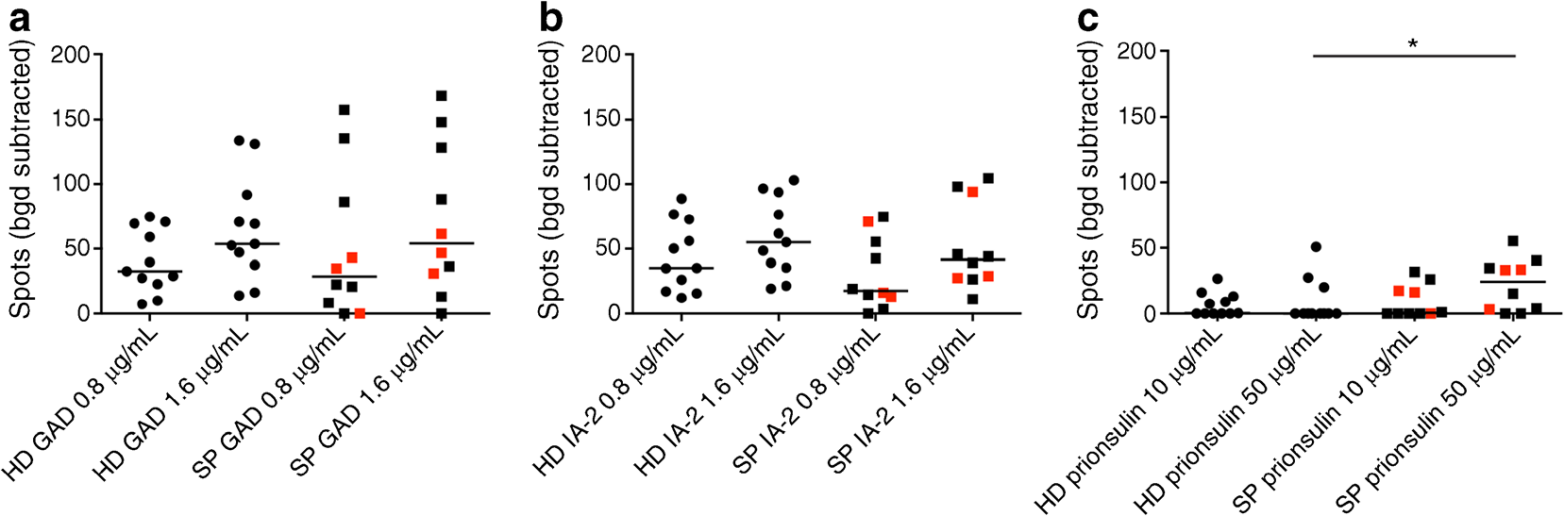
Islet autoantigen-specific B cell responses in slow progressors, measured by ELISpot, are increased to proinsulin but not GAD or IA-2. B cell responses to the indicated islet autoantigens were quantified using an ELISpot assay to detect secreted IgG. Background responses were determined in the absence of islet autoantigens. Results are shown as the number of background-subtracted spots per 4 × 10^5^ input PBMCs. Counts were averaged over at least six wells for each condition. ELISpot counts are shown for: (**a**) GAD at 0.8 μg/ml and 1.6 μg/ml; (**b**) IA-2 at 0.8 μg/ml and 1.6 μg/ml; (**c**) proinsulin at 10 μg/ml and 50 μg/ml. Horizontal lines indicate median values. Red squares denote slow progressors who tested seropositive for a single islet autoantibody at the time of immune cell analysis. *p < 0.05 for slow progressors vs healthy donors (determined using the Mann–Whitney *U* test). Bgd, background; HD, healthy donors; SP, slow progressors

Antibody-producing B cells specific for the islet autoantigens GAD, IA-2 and proinsulin were quantified using an ELISpot assay [22]. No significant differences were observed between slow progressors and healthy donors in response to stimulation with GAD or IA-2 (Fig. 6a,b). In contrast, slow progressors displayed higher frequencies of antibody-producing B cells in response to stimulation with proinsulin at 50 μg/ml (but not at 10 μg/ml), when compared with healthy donors (median number of spots was 24.34 in slow progressors vs 0 in healthy donors, *p* < 0.05) (Fig. 6c).

### Serum chemokines, cytokines and soluble receptors

No significant differences in serum levels of BAFF, CD95, CXCL9, CXCL10, CXCL11, IFN-γ, IL-4, IL-6, IL-10 or TGF-β were detected between slow progressors and healthy donors (data not shown). There was a non-statistically significant increase in soluble DcR3, the decoy receptor for CD95L (median serum concentration was 1087 pg/ml in slow progressors vs 651 pg/ml in healthy donors, *p* = 0.06) (ESM Fig. 6).

## Discussion

In this study, we conducted an extensive immunological analysis of slow progressors, individuals with newly diagnosed or long-standing type 1 diabetes, and healthy donors. Islet autoantibodies were lost over time in slow progressors. Moreover, various B cell subsets expressed higher levels of CD95 in slow progressors, especially after polyclonal stimulation, compared with the corresponding B cell subsets in healthy donors. These results suggested that autoreactive B cells may be more prone to activation-induced apoptosis and clonal deletion in individuals who are slow progressors, relative to those with newly diagnosed or long-standing type 1 diabetes and healthy donors.

Islet autoantigen-specific CD8^+^ T cell responses were largely absent in slow progressors and healthy donors. The phenotypic characteristics of CD4^+^ and CD8^+^ T cells were also similar in slow progressors and healthy donors. Slow progressors, however, exhibited decreased expression of CD95 among CD4^+^ T cells with a transitional memory phenotype (CD27^+^CD127^+^PD-1^++^), in contrast to most B cell subsets. In addition, lower frequencies of CD4^+^ T cells with a central memory phenotype (CD27^int^CD127^+^CD95^int^) were present in slow progressors compared with healthy donors, whereas higher frequencies of CD4^+^ T cells with a central memory phenotype (CD27^int^CD127^+^CD95^int^) were detected in people with newly diagnosed type 1 diabetes compared with healthy donors, consistent with an earlier report [23]. It is particularly intriguing that we detected low expression levels of CD95 among memory CD4^+^ T cells, because CD95 is known to promote apoptosis, which is required for the elimination of autoreactive T cells that escape thymic selection [24]. Previous reports have indicated that antigen-specific CD4^+^ T cells may be found in the peripheral blood of both healthy people and those with type 1 diabetes. The antigen-specific cells in individuals with type 1 diabetes expressed increased CXCR3 and decreased CCR7, indicating differentiation to Th1-like effector cells [25]. Further work will be required to examine the abundance and function of antigen-specific CD4^+^ T cells in slow progressors.

We, and others, have found previously that CD95 is expressed at relatively low levels on various B cell subsets in individuals with type 1 diabetes [6, 13]. We found the opposite in slow progressors. In individuals with systemic lupus erythematosus, B cells in general express relatively high levels of CD95, whereas autoreactive B cells in particular express relatively low levels of CD95 [26]. Moreover, disease flares have been associated with high frequencies of activated memory B cells, defined by the expression of CD95 [26, 27]. Serum levels of DcR3, the decoy receptor for CD95L, increased from a median of 651 pg/ml in healthy donors to 1087 pg/ml in slow progressors (*p* = 0.06). Therefore, the changes in expression of CD95 could also suggest that there is increased activity of autoreactive B cells (unsurprising as the BOX participants are defined by the production of autoantibodies).

We reported previously that while B cells from healthy individuals can respond to pancreatic antigens, including GAD and IA2 when measured using an ELISpot assay, the B cells from people with type 1 diabetes have a significantly increased response [22]. Here we demonstrate that slow progressors do not respond significantly more than healthy donors to either GAD or IA2 but have significantly more spot-forming cells in response to proinsulin. The lack of increased response to GAD and IA2 was interesting, given that many of the slow progressors were positive for GAD autoantibodies in particular. In addition, the polyclonal pre-stimulation induces memory B cells to differentiate into antibody secreting cells [28] and would be expected to overcome any anergy present in autoreactive B cells. Thus, we infer that GAD- and IA2-reactive B cells are not more abundant in the peripheral blood of the slow progressors compared with healthy donors and suggest that the presence of autoantibodies in the serum may be due to lower thresholds of B cell stimulation for antibody production in the slow progressors. We also manually gated for CD24^+++^CD38^++^ ‘Bregs’ and found no difference between the healthy donors and slow progressors (data not shown). No direct comparison was made with individuals newly diagnosed with type 1 diabetes due to the inability to age-match slow progressors and newly diagnosed individuals, the latter developing diabetes at a younger age.

One limitation of this study was the lack of a direct comparator group, namely at-risk individuals who subsequently progressed to type 1 diabetes. Moreover, immune cell variables were measured after some islet autoantibodies had already been lost, potentially impacting the detection of autoreactive B cell responses to GAD and IA-2. It would be informative to study immune profiles in slow progressors before and after islet autoantibody loss. In the small cohort that we studied, we did not observe differences when comparing the slow progressors who were only positive for a single autoantibody at the time of sampling (indicated in red symbols throughout the figures) and those who retained multiple autoantibody positivity; this would require repetition with more slow progressors in order for firm conclusions to be drawn. It is also possible that we selected for a particular subgroup of at-risk individuals, defined by the presence of islet autoantibodies, while another subgroup may have more autoreactive T cells. Additional studies are therefore required to confirm the findings reported here. Our data nonetheless reveal a mechanistically coherent pattern of immunological traits in slow progressors, suggesting a greater propensity for apoptotic regulation of peripheral autoimmunity compared with healthy individuals.

We are aware that the number of suitable BOX participants that we used here is small and we aim to validate and expand upon the results described here by studying slow progressors from other collections in the SNAIL project.

In summary, B and T cells from slow progressors were very similar to those from healthy individuals. Crucially, CD8^+^ T cell response to diabetogenic peptides were similarly low in slow progressors and healthy individuals and we did not observe an expansion of GAD or IA-2 antibody-producing B cells in the peripheral blood. However, changes in CD95 on B cells in slow progressors suggested the possibility of increased susceptibility of B cells to apoptosis. In future studies, we will aim to increase cohort sizes in order to address the functional roles of the phenotypic changes, and to identify markers predictive of protection from disease.

## Electronic supplementary material

ESM (PDF 15333 kb)

## Data Availability

The datasets generated and/or analysed during the current study are available from the corresponding author on reasonable request.

## Abbreviations

BAFF: B cell activating factor
BOX: Bart’s Oxford Family Study
CXCR3: C-X-C motif chemokine receptor 3
DcR3: Decoy receptor 3
ELISpot: Enzyme-linked immunospot
GADA: GAD autoantibodies
IA-2: Islet antigen-2
IA-2A: Islet antigen-2 autoantibodies
IAA: Insulin autoantibodies
PBMC: Peripheral blood mononuclear cell
PD-1: Programmed cell-death protein 1
SNAIL: Slow or Non-progressive Autoimmunity to the Islets of Langerhans
SPADE: Spanning-tree Progression Analysis of Density-normalized Events
ZnT8A: Zinc transporter 8 autoantibodies
