# Supplementary material for: Slow progressors to type 1 diabetes lose islet autoantibodies over time, have few islet antigen-specific CD8+ T cells and exhibit a distinct CD95hi B cell phenotype
Source: Diabetologia. 2020 Mar 10;63(6):1174–85. doi: 10.1007/s00125-020-05114-7 (PMC7228996; doi:10.1007/s00125-020-05114-7)
Supplement: Supplementary file 1 — (PDF 15333 kb) [file 125_2020_5114_MOESM1_ESM.pdf]

Electronic Supplementary Material (ESM)

Slow progressors to type 1 diabetes lose islet autoantibodies over time, have few islet antigen-specific CD8^{+} T cells and exhibit a distinct CD95^{hi} B cell phenotype

Stephanie J. Hanna[1], Wendy E. Powell[1], Anna E. Long[2], Emma J. S. Robinson[1], Joanne Davies[1], Clare Megson[2], Alexandra Howell[1], Taz J. Jones[1], Kristin Ladell[1], David A. Price[1], Colin M. Dayan[1], Alistair J. K. Williams[2], Kathleen M. Gillespie[2] and F. Susan Wong[1]

1. Division of Infection and Immunity, Cardiff University School of Medicine, Heath Park, Cardiff CF14 4XN, UK
2. Diabetes and Metabolism, Bristol Medical School, University of Bristol, Bristol, UK

Stephanie J. Hanna, Wendy E. Powell, Anna E. Long and Emma J. S. Robinson contributed equally to this work. Kathleen M. Gillespie and F. Susan Wong are joint senior authors.

Abbreviations

HD	healthy donors
LS	long-standing type 1 diabetes
ND	newly diagnosed type 1 diabetes
SP	slow progressors

ESM Table 1

a

	Number	Mean age (yrs)	Age range (yrs)	Mean disease duration (yrs)	Range of disease duration (yrs)
HD male	14	34.6	18–72	-	-
HD female	9	34.2	20–55	-	-
SP male	6	34.5	28–69	-	-
SP female	4	43.3	33–55	-	-
ND male	12	31.3	19–45	0.4	0.1–1.0
ND female	6	29.0	18–50	0.4	0.1–0.9
LS male	10	38.4	20–48	23.1	3–38
LS female	8	34.1	18–49	18.9	3–36

b

	Gender	Age (years)	HLA Class II DRB1 genotyping		GADA (DK units/ml)	IA-2A (DK units/ml)	ZnT8A (units/ml)	IAA (units/ml)
HD1	M	52	*0701-0702	*0901	4.15	0.00	0.41	0.00
HD2	M	26	*0401-0422	*0805-0801	0.00	0.00	0.49	0.00
HD3	M	34	*0401-0422	*1302-1305/1303-1304	0.00	0.00	0.34	0.00
HD4	M	37	*0701-0702	*1001	0.33	0.00	0.33	0.00
HD5	M	72	*0101-0103	-	0.00	0.00	1.01	0.00
HD6	M	41	*0401-0422	*0701-0702	0.00	0.00	0.49	0.00
HD7	M	18	*1501-1505	*0701-0702	0.00	0.00	0.46	0.00
HD8	M	29	*0401-0422	*1302-1305/1303-1304	0.00	0.00	0.31	0.00
HD9	M	34	*1501-1505	*0701-0702	24.53	0.00	1.01	0.00
HD10	M	29	*0301	-	0.00	0.00	0.21	0.00
HD11	M	22	*1101-1121	*1302-1305/1303-1304	0.00	0.00	0.29	0.00
HD12	M	21	*1501-1505	-	0.00	0.00	0.40	0.00
HD13	M	24	*0401-0422	*0701-0702	0.00	0.00	0.45	0.00
HD14	M	46	*0101-0103	*0301	4.49	0.00	0.29	0.00
HD15	F	21	*0701-0702	-	0.00	0.00	0.32	0.00
HD16	F	49	*0301	*0701-0702	0.00	0.00	0.39	0.00
HD17	F	24	*1501-1505	*0701-0702	0.00	0.00	0.43	0.00
HD18	F	20	*0301	*0701-0702	0.00	0.00	0.72	0.00
HD19	F	38	*0101-0103	*0401-0422	0.00	0.00	0.54	0.00
HD20	F	44	*0401-0422	*1101-1121	nk	nk	nk	nk
HD21	F	33	*0301	*0805-0801	0.00	0.00	0.15	0.00
HD22	F	55	*1302-1305/1303-1304	-	0.00	0.00	0.45	0.00
HD23	F	24	*0401-0422	-	nk	nk	nk	nk
SP1	M	28	*0101-0103	*0301	309.30	0.00	0.38	0.00
SP2	M	41	*0301	*0401-0422	176.00	8.20	6.70	0.00
SP3	M	69	*0401-0422	*1201-1203	82.25	0.00	92.86	0.00
SP4	M	53	*0401-0422	*0701-0702	715.30	5.96	0.44	0.82
SP5	M	46	*0401-0422	*1501-1505	55.01	0.00	0.47	0.00
SP6	M	31	*0301	*0401-0422	146.59	0.00	4.34	0.00
SP7	F	33	*0101-0103	*0301	267.26	0.00	0.70	0.00
SP8	F	44	*0401-0422	*1101-1121	205.75	25.74	11.88	0.00
SP9	F	55	*0101-0103	*0401-0422	456.13	0.00	0.25	0.30
SP10	F	41	*0401-0422	*1001	362.95	228.42	7.13	0.00

c

	Gender	Age (years)	Disease duration (yrs)	HLA Class II *DRB1* genotyping		GADA (DK units/ml)	IA-2A (DK units/ml)	ZnT8A (units/ml)
LS1	M	48	32.0	**0301*	**0401-0422*	122.21	106.47	13.91
LS2	M	33	3.0	**0301*	-	183.84	81.25	0.47
LS3	M	39	30.0	**0301*	**0401-0422*	13.48	0.00	0.44
LS4	M	20	13.0	**0101-0103*	**0301*	1.73	0.00	0.29
LS5	M	35	22.0	**0301*	**0701-0702*	51.32	0.00	0.39
LS6	M	29	13.0	**0301*	-	67.57	239.50	41.41
LS7	M	48	36.0	**0401-0422*	-	175.45	0.00	0.88
LS8	M	45	31.0	**0401-0422*	**0701-0702*	3.00	0.00	0.65
LS9	M	43	13.0	**0101-0103*	**0301*	5.14	1.74	0.88
LS10	M	44	38.0	**0101-0103*	**0401-0422*	0.00	0.00	0.31
LS11	F	33	17.0	**0101-0103*	**0401-0422*	86.75	0.00	0.37
LS12	F	44	14.0	**0301*	-	900.07	0.00	0.36
LS13	F	34	31.0	**0401-0422*	**1302-1305/1303-1304*	0.00	0.08	0.49
LS14	F	37	13.0	**0401-0422*	**1101-1121*	79.94	2.10	0.24
LS15	F	34	25.0	**0301*	**0401-0422*	1.51	3.31	0.95
LS16	F	24	12.0	**0301*	**0401-0422*	5.37	0.00	0.41
LS17	F	49	36.0	**0301*	**0401-0422*	16.61	0.00	0.52
LS18	F	18	3.0	**0301*	**0401-0422*	1,018.96	0.00	0.51
ND1	M	36	0.3	**0401-0422*	-	11.68	0.00	0.25
ND2	M	28	0.1	**1501-1505*	**0401-0422*	0.00	0.00	0.46
ND3	M	36	0.3	**0401-0422*	**1302-1305/1303-1304*	221.12	271.26	0.71
ND4	M	27	0.3	**0401-0422*	-	193.87	29.82	0.41
ND5	M	37	0.1	**0101-0103*	**0301*	869.57	267.67	126.84
ND6	M	34	1.0	**0101-0103*	**0401-0422*	15.31	0.00	0.50
ND7	M	45	0.9	**0301*	**0701-0702*	69.99	201.51	0.54
ND8	M	21	0.4	**1501-1505*	-	0.00	0.00	0.21
ND9	M	24	0.7	**0301*	**0401-0422*	314.61	124.11	0.28
ND10	M	24	0.2	**0101-0103*	**0701-0702*	345.06	0.00	0.49
ND11	M	45	0.3	**0301*	**0701-0702*	458.91	0.00	0.57
ND12	M	19	0.3	**0301*	**0701-0702*	1,015.09	300.97	0.41
ND13	F	18	0.3	**0301*	**0401-0422*	0.00	2.54	69.83
ND14	F	50	0.7	**0301*	**0401-0422*	0.00	0.00	1.97
ND15	F	23	0.3	**0401-0422*	-	500.65	280.68	106.96
ND16	F	23	0.9	**0301*	**0401-0422*	517.54	0.00	0.26
ND17	F	36	0.3	**0301*	**0701-0702*	663.30	110.65	0.68
ND18	F	24	0.1	**0401-0422*	**1302-1305/1303-1304*	173.03	3.68	4.30

ESM Table 1. Clinical, demographic, and immunological characteristics of SPs, people with ND or LS type 1 diabetes, and HDs. (**a**) Summary table of demographics (**b**) HDs' and SPs' demographic and immunological characteristics (**c**) Clinical, demographic, and immunological characteristics of people with newly diagnosed or long-standing type 1 diabetes**.** nk: not known. SP6 had autoimmune thyroid disease. Thresholds for seropositive islet autoantibody titres: GADA ≥ 33 DK units/ml; IA-2A ≥ 1.4 DK units/ml; ZnT8A ≥ 1.8 units/ml; IAA ≥ 0.2 units/ml. HD, healthy donors; LS, long-standing type 1 diabetes; ND, newly diagnosed type 1 diabetes; SP, slow progressors

ESM Table 2

Peptide	Sequence	QDot 1	QDot 2	Panel
CMV	NLVPMVATV	585	800	A
EBV	GLCTLVAML	655	800	A
non-interfacing	GLFGGGGGV	585	605	A
INS b10-18	HLVEALYLV	605	655	B
PI 15-24	ALWGPDPAAA	585	655	B
GAD 114-123	VMNILLQYVV	655	800	B
IA-2 797-805	MVWESGCTV	585	605	B
IGRP 265-273	VLFGLGFAI	605	800	B
ppIAPP 5-13	KLQVFLIVL	585	800	B

ESM Table 2. Peptide-HLA-A2 tetramers used in this study.

ESM Figure 1

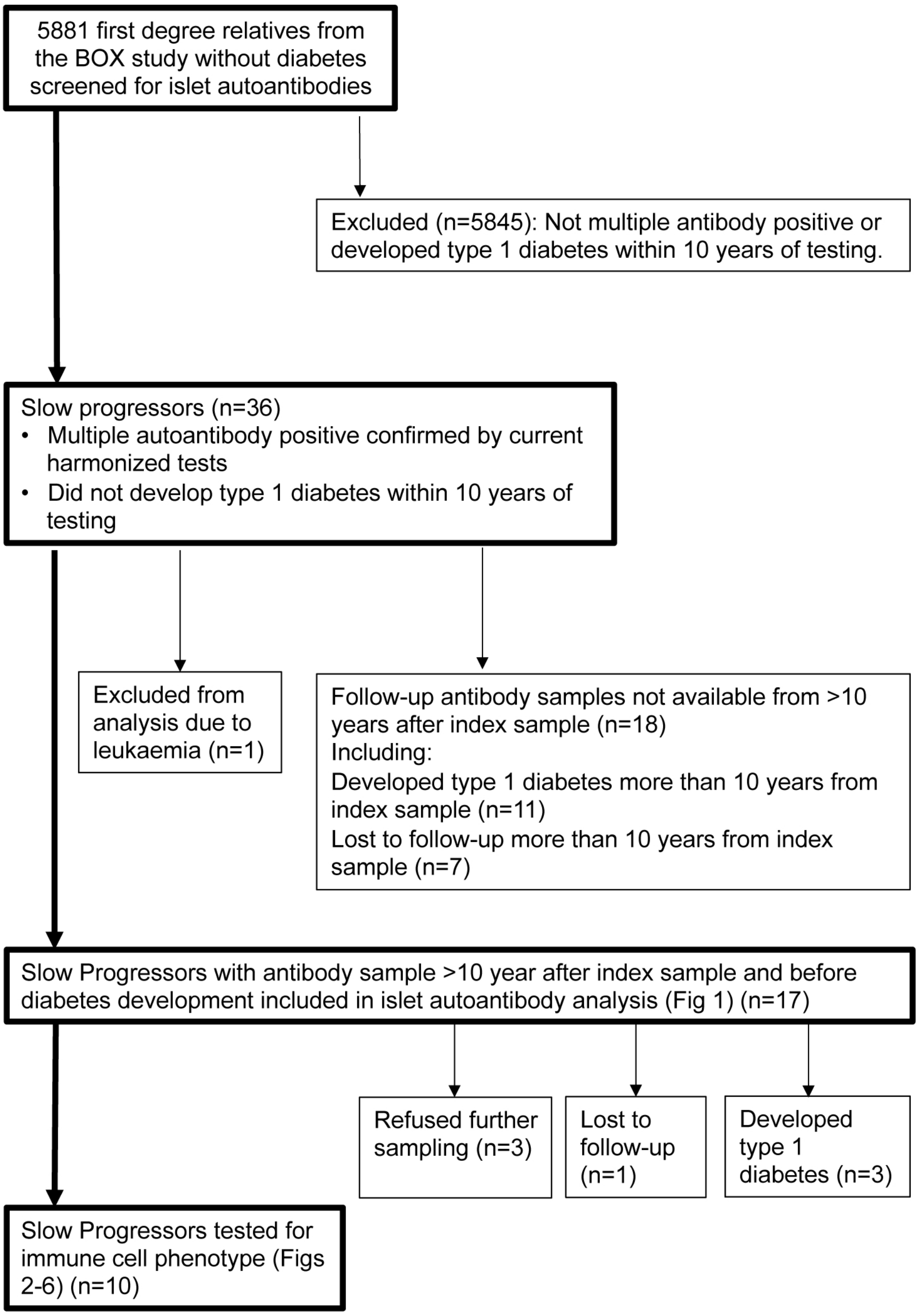

ESM Figure 1: Consort diagram of slow progressors.

ESM Figure 2

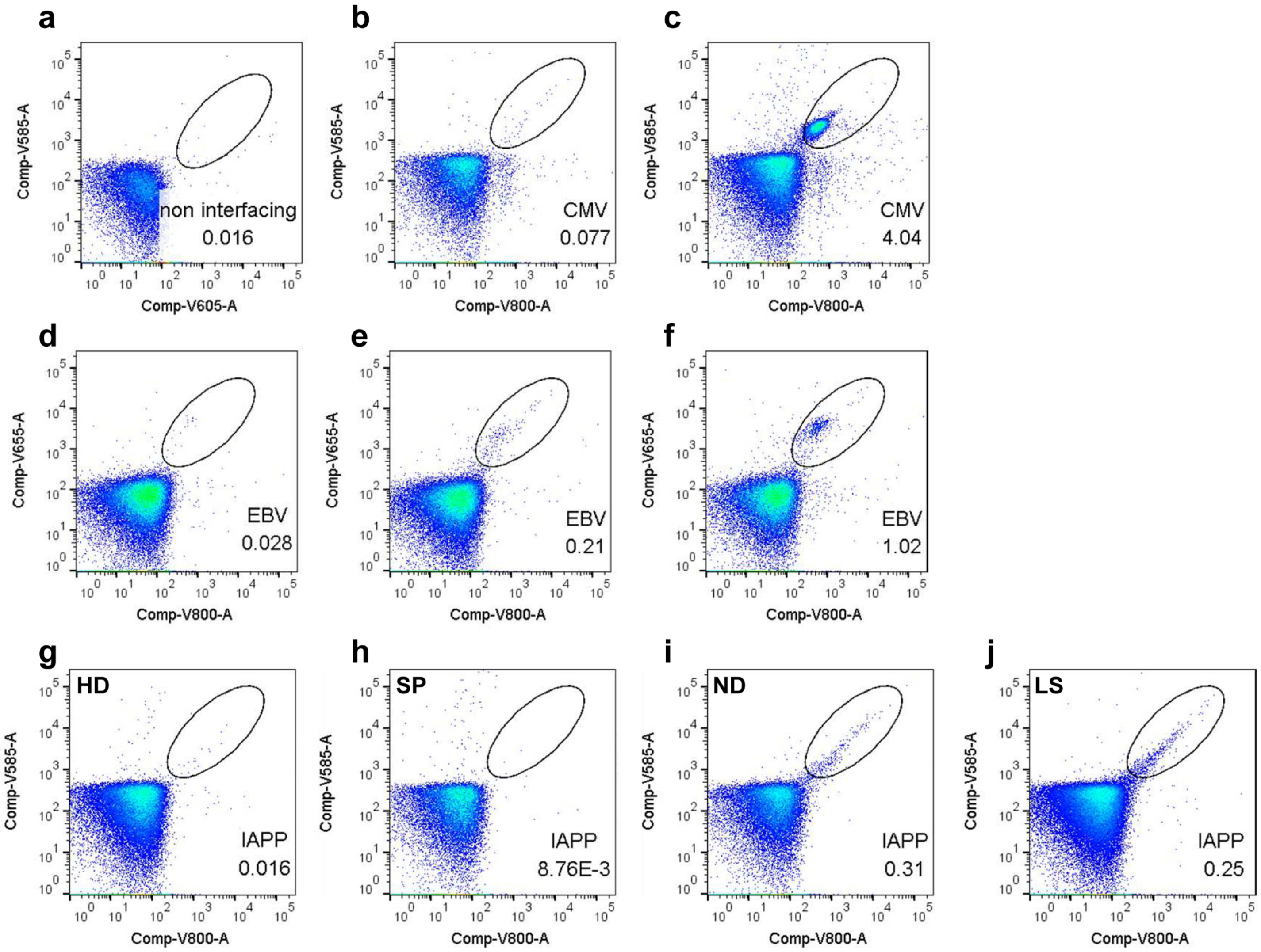

ESM Figure 2: Representative flow cytometric plots from negative and positive control data in Figure 2, illustrating different levels of response: a. staining with non-interfacing peptide tetramer; b, c. staining with CMV peptide tetramer; d,e,f staining with EBV peptide tetramer; g. staining with IAPP peptide tetramer from HD; h. staining with IAPP peptide tetramer from SP; i. staining with IAPP peptide tetramer from ND; j. staining with IAPP peptide tetramer from LS. HD, healthy donors; LS, long-standing type 1 diabetes; ND, newly diagnosed type 1 diabetes; SP, slow progressors

ESM Figure 3

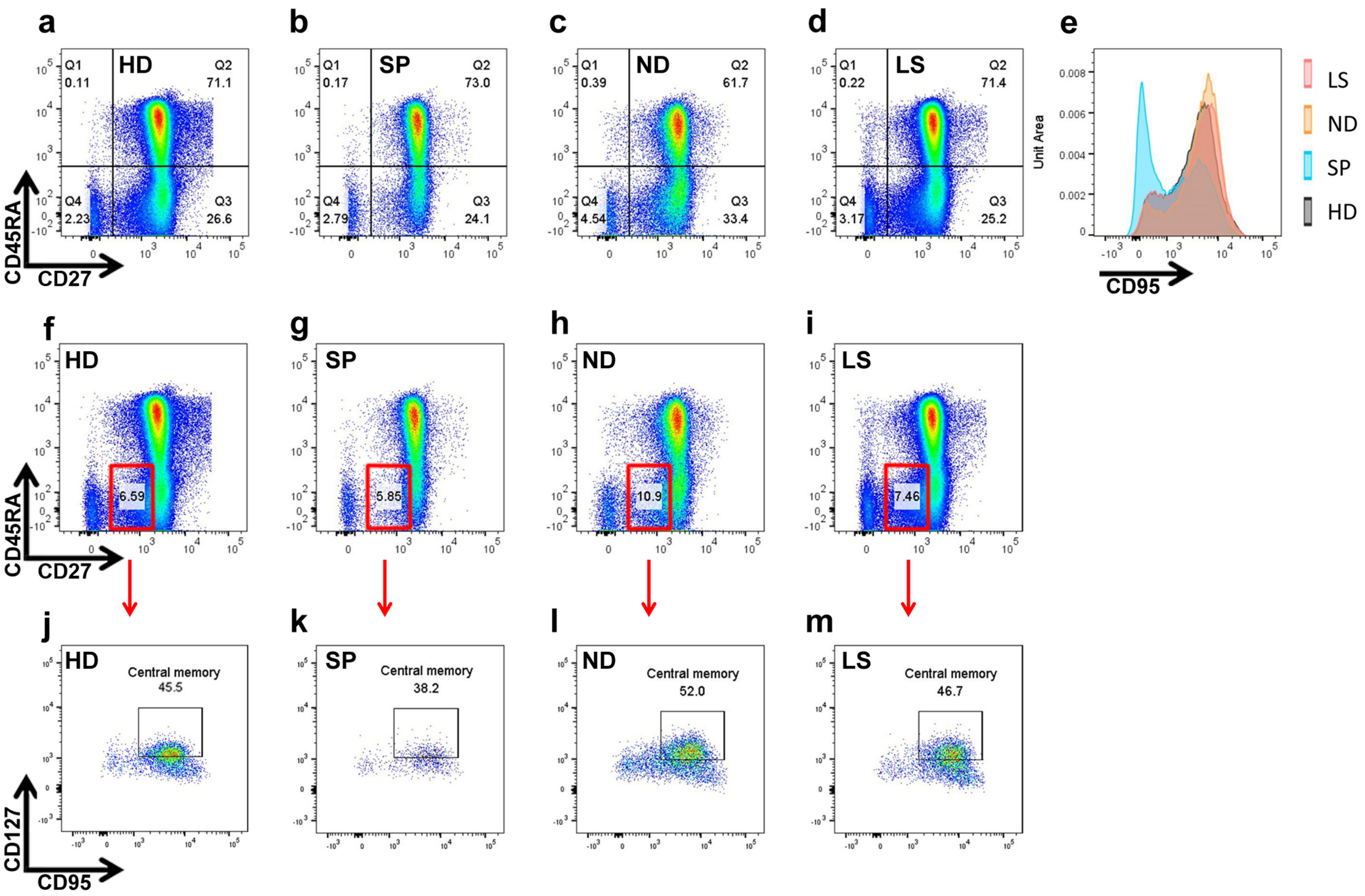

ESM Figure 3: Representative flow cytometric plots from data in Figure 3. Quadrant-gated CD4^{+} T cells a. HD; b. SP; c. ND; d. LS; e. histogram overlay of CD95 expression in Q3 of the quadrant gated plots (CD27^{+}CD45RA^{-} T cells). f-i CD4^{+} plots with a CD27^{int} CD45RA^{-} population box-gated manually in FlowJo f. HD; g. SP; h. ND; i. LS; j-m CD127 and CD95 expression in the box gated populations (parts f-i), indicating the central memory population that is analogous to cells shown in Fig 3d of the main text; j. HD; k. SP; l. ND; m. LS. HD, healthy donors; LS, long-standing type 1 diabetes; ND, newly diagnosed type 1 diabetes; SP, slow progressors

ESM Figure 4

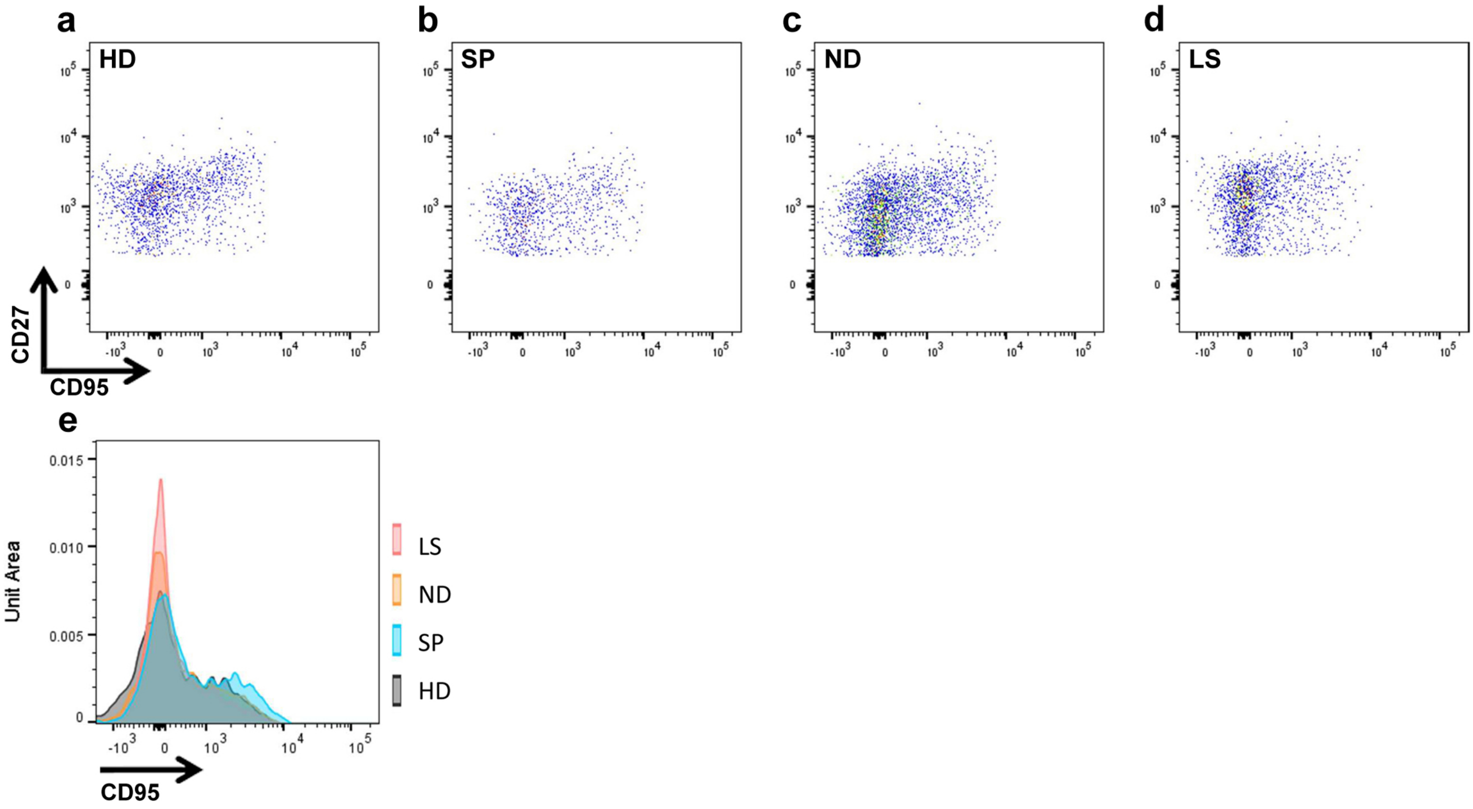

ESM Figure 4: Representative flow cytometric plots from data in Figure 4. Switched memory B cells ($CD27^{+}IgD^{-}$) from freshly isolated PBMC, manually gated in FlowJo, showing expression of CD27 and CD95: a. HD; b. SP; c. ND; d. LS; e. a histogram of CD95 expression in the switched memory B cells. HD, healthy donors; LS, long-standing type 1 diabetes; ND, newly diagnosed type 1 diabetes; SP, slow progressors

ESM Figure 5

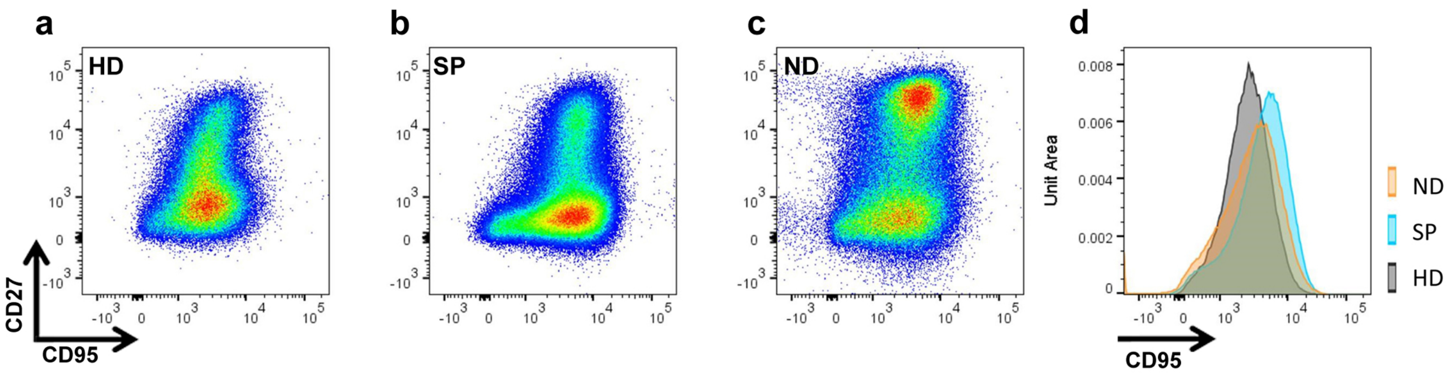

ESM Figure 5: Representative flow cytometric plots from data in Figure 5. B cells following *ex vivo* stimulation showing expression of CD27 and CD95: a. HD; b. SP; c. ND; d. histogram overlay of CD95 expression in the switched memory B cells. HD, healthy donors; ND, newly diagnosed type 1 diabetes; SP, slow progressors

ESM Figure 6

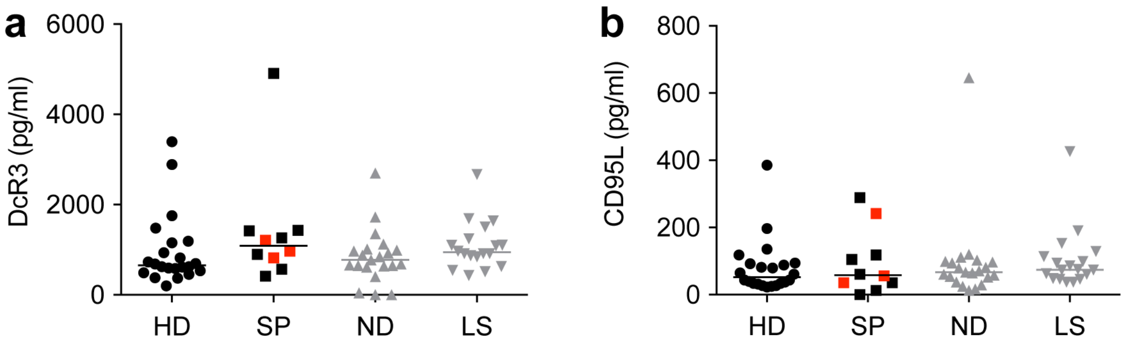

ESM Figure 6: Serum levels of DcR3 and CD95L. Serum concentrations of DcR3 and CD95L were measured using ELISAs. ELISA results are shown: a. DcR3; b. CD95L. Horizontal lines indicate median values. Red squares denote slow progressors who tested seropositive for a single islet autoantibody at the time of immune cell analysis. HD, healthy donors; LS, long-standing type 1 diabetes; ND, newly diagnosed type 1 diabetes; SP, slow progressors
